# Finite element analysis modeling of plates versus intramedullary nails in closed comminuted midshaft tibial fractures

**DOI:** 10.1051/sicotj/2022025

**Published:** 2022-06-16

**Authors:** Mahmoud Ahmed El-Desouky, Ayman Ali Saleh, Sherif Mamdouh Amr, Ahmed Samir Barakat

**Affiliations:** 1 Department of Orthopedic Surgery and Traumatology, Faculty of Medicine, Cairo University 11562 Cairo Egypt; 2 Department of Orthopedic Surgery and Traumatology, Aseer Central Hospital 62523 Abha Saudi Arabia

**Keywords:** Tibial fracture, Interlocking nail, Locking plate, Finite element analysis

## Abstract

*Background*: Tibial shaft fractures are usually treated by interlocking nails or plates. The ideal implant choice depends on many variables. *Aim*: To assess the mechanical behavior of interlocking nails and plates in the treatment of closed comminuted midshaft fractures of the tibia using finite element analysis. *Material and methods*: This is a prospective study of 50 patients with a mean age of 28.4 years with closed comminuted fractures of the midshaft of the tibia. Data evaluation was done by Finite element analysis (FEA). Fixation was revised in two cases. *Results*: After load application, there were significant differences in both bending (*P* = 0.041) and strain percent (*P* = 0.017), reflecting that interlocking nails were superior to plates. There were also significant differences between titanium and stainless-steel materials in bending (*p* = 0.041) and strain percent (*p* = 0.017) after applying load, indicating that titanium was superior to stainless steel. *Conclusion*: Interlocking nails are superior to plates in treating midshaft tibial fractures. The use of blocking screws may be needed in interlocking nails depending on the pattern and extension of the fracture.

## Introduction

Tibial fractures represent about 36.7% of all long-bone fractures in adults. They usually result from high energy injuries, especially in road traffic accidents. Since almost one-third of the tibial surface is located subcutaneously, overlying soft tissue injuries are commonly encountered, and open tibial fractures account for over 40% of all open fractures [1, 2].

Tibial fractures can cause extensive disability and prolonged morbidity unless treated properly [3]. Treatment modalities of tibial shaft fractures vary and are based on the fracture pattern, soft tissue injuries, patient age, presence of co-morbidities, and bone density [4]. Interlocking intramedullary nailing (IMNs), various plate designs, and external fixator devices have universally been described [5–7].

Finite element analysis (FEA) is extensively used in engineering to assess the mechanical and microstructural behavior of structures when subjected to predefined loads to confirm their integrity prior to manufacture [8]. FEA has been used by several researchers to predict the bone structure behavior with implants under certain loading conditions [9].

Although interlocking nail devices are the generally accepted implant of choice in closed comminuted midshaft fractures of the tibia due to their better clinical outcome and lower complication rates compared to plates, it may be interesting to compare the biomechanical properties of both devices based on finite element analysis (FEA). This prospective study aims to compare the use of the interlocking intramedullary nail and locking plate and screws for the surgical treatment of closed comminuted fractures of the tibial shaft using the Finite element analysis.

## Materials and methods

This is a prospective comparative study of 50 patients with closed comminuted fractures of the shaft of the tibia who had fixation using either interlocking nails (IMNs) or locking plates (LPs). The data were collected consecutively from April 2019 to October 2020 after obtaining the Ethical board approval from our institution.

This work was conducted in accordance with the ethical standards of the Helsinki Declaration. All participating patients signed an informed consent regarding the procedure, possible complications, and alternative treatment modalities. No experiments on animals were conducted.

Our study included 50 patients with a mean age of 28.4 ± 8.3 years old and a mean weight of 66.7 ± 20.1 kg. Out of 50 patients, 41 (82.0%) were males, and 9 (18.0%) were females. Twenty-three patients (46.0%) had right-side affection, and the other 27 (54.0%) had left-side affection. Fractures were encountered following road traffic accidents (RTA) in 41 cases (82.0%), falling from a height (FFH) in 7 cases (14%), and falling downstairs in 2 cases (4.0%).

Patients enrolled in the study were randomized by the even/odd numbers technique into two groups. Half of them (*n* = 25) had fixation using interlocking nails (ILNs), and the other half (*n* = 25) had fixation using locking plates (LPs) and screws. Fixation was revised in two cases using IMNs. So, the study included 52 cases, 27 cases (51.9%) with IMN fixation and 25 cases (48.1%) with LP fixation. Titanium was the most commonly used fixation material in 39 cases (75.0%), whereas stainless steel was used in 13 cases (25.0%).

### Implants

The interlocking nails used in the cases included Tibial interlocking nail-Zimmer Natural Nail System (Zimmer), TN-Advanced Tibial Nailing System (DepuySynthes), and ZTN Tibial Nail (ZIMED). While the plates used included Synthes Locking compression Plate, Narrow and Broad (DepuySynthes), or Locking Compression Plates (JMS). The choice of the type of nail or plate was according to the availability at the time of surgery.

### Finite element modeling

Immediate postoperative radiographs in anteroposterior (AP) and lateral planes were subjected to Finite element analysis (FEA) using COSMO/M-Finite Element Analysis Software 2.7 (Structural Research and Analysis Corporation (SRAC), Santa Monica, California, USA). The employed three-dimensional (3-D) “lower leg” finite element model (LLFE model) included the tibia and the fibula. The bony structures were generated by the segmentation of data obtained from X-rays by the Visible Human Project (National Library of Medicine (NLM), USA). The 3-D models of the interlocking nails and locking plates with screws were designed using computer-aided design (CAD) according to the configurations of the used implants. The maximum load was determined by the formula: bodyweight + 0.7 × bodyweight = maximum load in kilograms (kg). The displacement ratio at the minimum fracture gap after applying the load was calculated, thus identifying the strain percent in the gap after applying the load.

### Statistical analysis

The collected data were coded and then transferred to SPSS software version 24 (IBM, Newark, NY, USA) for data analysis. Descriptive statistics as numbers and frequencies were used to describe the qualitative variables, whereas quantitative variables were represented using mean and standard deviation. The relationships between different variables were tested using chi-square analysis (*χ*^2^) or *t*-student test according to the type of data. A *P*-value of ≤ 0.05 was considered statistically significant.

## Results

In this study, the maximum load applied ranged from 700 to 2230 N, with a mean value of 1425 ± 471.5 N. The range of the current minimum fracture gap was 1.5-5 mm, with a mean value of 2.4 ± 0.9 mm. The bending range was 0.012–1.08 mm, with a mean value of 0.214 ± 0.231 mm, and a median of 0.151 mm. The range of the strain percent in the gap after applying the load was 0.78–36, with a mean value of 8.214 ± 7.675.

### Correlations between the investigated parameters and sex

There was no significant difference between males and females regarding the current minimum fracture gap, resultant bending, and the strain percent after applying the load (*p* = 0.156, 0.610, 0.202, respectively).

### Correlations between the investigated parameters and age

Neither the current minimum fracture gap nor the strain percent in the gap after applying the load was significantly correlated with age (*p* = 0.124 and 0.110, respectively). A significant negative correlation was present between bending and age (*p* = 0.050) (Table 1).

**Table 1:** Investigated correlations.

Variables		Age (years)	Weight (kg)	Maximum load applied (*N*)
Current minimum fracture gap (mm)	*r*	−0.338	0.379	0.332
	*p*	0.124	0.082	0.132
Resultant bending (mm)	*r*	−**0.423**	0.102	−0.193
	*p*	**0.050**	0.651	0.388
The strain percent (%) in the gap after applying the load	*r*	−0.350	−0.104	−0.416
	*p*	0.110	0.645	0.054

Values in bold are the statistically significant results.

### Correlations between the investigated parameters and patients’ weights

Weight had no significant correlation with either the current minimum fracture gap, bending, or the strain percent in the gap after load application (*p* = 0.082, 0.651, 0.645, respectively) (Table 1).

### Correlations between the investigated parameters and maximum load applied

**Figure 1:**
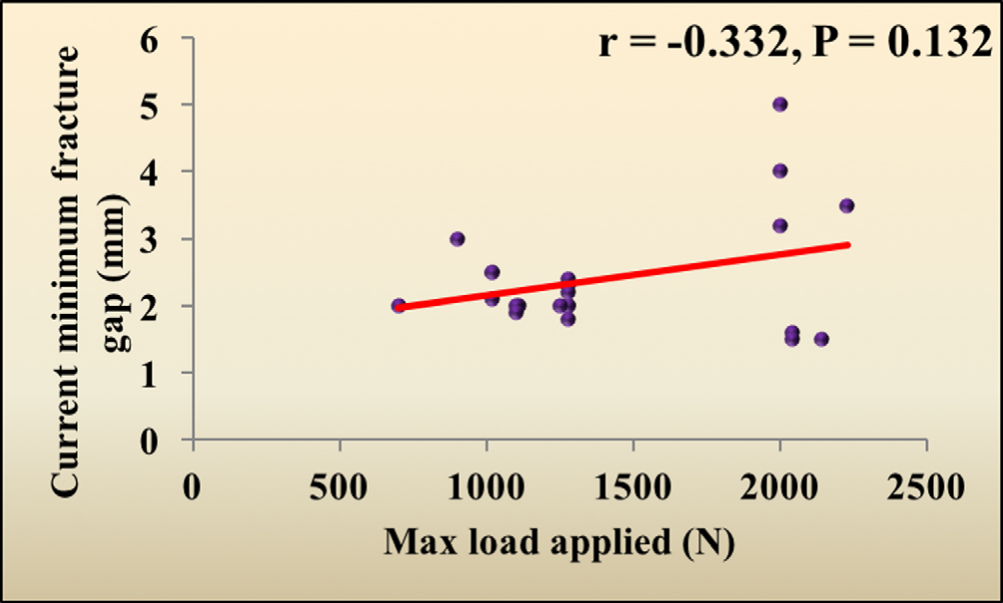
The correlation between the maximum load applied and the current minimum fracture gap.

**Figure 2:**
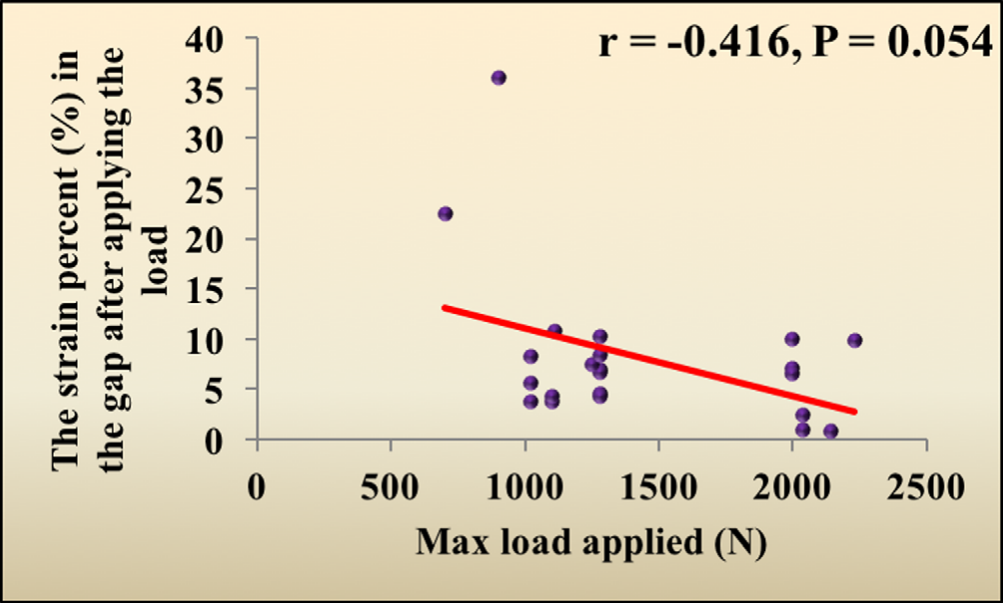
The correlation between the maximum load applied and the strain percent in the gap after applying the load.

The maximum load applied had no significant correlation with either the current minimum fracture gap, bending, or the strain percent in the gap after load application (*p* = 0.132, 0.388, 0.054, respectively) (Table 1) (Figures 1 and 2).

### Comparison of device materials (titanium and stainless steel)

There was no significant difference between the rod materials titanium and stainless steel in the current minimum fracture gap (*p* = 0.636). Regarding bending, stainless steel significantly had higher resultant bending compared to titanium (*p* = 0.041). There was a significant difference in the strain percent after applying the load between stainless steel and titanium (*P* = 0.017) (Table 2).

**Table 2:** The correlations between the rod material and procedures measures.

	Fixation rod material		
Variables	Titanium (*n* = 39)	Stainless steel (*n* = 13)	*P*-value
Current minimum fracture gap (mm)			0.636
Range	1.5–5	2–3	
Mean ± SD	2.4 ± 0.9	2.3 ± 0.5	
Median (IQR)	2 (1.9–2.5)	2.2 (2.1–2.6)	
Resultant bending (mm)			**0.041**
Range	0.012–0.5	0.174–1.08	
Mean ± SD	0.157 ± 0.122	0.473 ± 0.425	
Median (IQR)	0.139 (0.08–0.216)	0.318 (0.18–0.765)	
The strain percent after applying the load			**0.017**
Range	0.78–10.8	8.28–36	
Mean ± SD	5.859 ± 3.079	18.808 ± 13.258	
Median (IQR)	6.024 (3.79–7.49)	15.475 (8.365–29.25)	

Values in bold are the statistically significant results.

### Comparison of plates and intramedullary nails

There was no significant difference between the interlocking nail and locking plate regarding the current minimum fracture gap (*p* = 0.636). But plates had significantly higher resultant bending and strain percent after load application compared to nails (*p* = 0.041, 0.017, respectively) (Table 3).

**Table 3:** The correlations between the type of implant and procedures measures.

	Implant		
Variables	Interlocking nails (*n* = 27)	Locking plates (*n* = 25)	*P*-value
Current minimum fracture gap (mm)			0.636
Range	1.5–5	2–3	
Mean ± SD	2.4 ± 0.9	2.3 ± 0.5	
Median (IQR)	2 (1.9–2.5)	2.2 (2.1–2.6)	
Resultant bending (mm)			**0.041**
Range	0.012–0.5	0.174–1.08	
Mean ± SD	0.157 ± 0.122	0.473 ± 0.425	
Median (IQR)	0.139 (0.08–0.216)	0.318 (0.18–0.765)	
The strain percent after applying the load			**0.017**
Range	0.78–10.8	8.28–36	
Mean ± SD	5.859 ± 3.079	18.808 ± 13.258	
Median (IQR)	6.024 (3.79–7.49)	15.475 (8.365–29.25)	

Values in bold are the statistically significant results.

## Discussion

The peak incidence of tibial fractures occurs among young males. It accounts for 21.5/100,000 patients-year for males versus 12.3/100,000 patients-year for females, and the mean age of injury for men is 38.5 years [1].

In this study, a biomechanical assessment of the behavior of fixation devices made from different materials was performed through finite element modeling. Immediate postoperative radiographs in anteroposterior (AP) and lateral planes were subjected to finite element analysis (FEA). The mean maximum load applied was 1425 N. The mean current minimum fracture gap was 2.4 mm. The mean resultant bending was 0.214 mm. The mean strain percent in the gap after load application was 8.214.

Plates had significantly higher resultant bending and strain percent after load application compared to intramedullary nails (*p* = 0.041, 0.017, respectively), while there was no significant difference between both regarding the current minimum fracture gap (*p* = 0.636). Titanium had significantly lower resultant bending and lower strain after load application compared to stainless steel (*p* = 0.041 and 0.017, respectively).

Titanium and stainless steel are the commonly used materials to manufacture the fixation rods used to treat tibial shaft fractures [10]. In the current study, titanium was used for 75% of the cases, whereas stainless steel was used for 25%. Titanium significantly showed lower bending (0.157 mm vs. 0.473 mm) and strain percent (5.859 vs. 18.808) after applying load compared to stainless steel underlining the superiority of titanium.

Hauke et al., in their study, found that the infection rate with titanium was lower than that with stainless steel (59% vs. 82%) [10]. Arens et al. also reviewed the relationship between the infection rates and plate materials and found a higher infection rate associated with the use of stainless steel plates compared with titanium [11]. Vinchhi et al. conducted a study on 45 patients with fracture tibia, with 31 patients treated with titanium nails and 14 patients with stainless steel nails. Two patients (6.45%) treated with titanium nails and 2 patients (14.29%) treated with stainless steel nails had infections [12].

Interlocking IMN is considered the treatment of choice for most tibial shaft fractures as it is associated with negligible blood loss, minimal surgical trauma, and less non-union. It allows earlier weight-bearing and a shorter hospital stay [13]. Whereas indications for the plate in treating tibial shaft fracture are limited [14].

Our study observed significant differences in resultant bending and strain percent after applying the load between interlocking nails and plates (*p* = 0.041, 0.017, for bending and percent strain, respectively). The mean bending was significantly higher in plates (mean = 0.473 mm) compared to interlocking nails (mean = 0.157 mm), and the mean of strain percent was significantly higher in plates (mean = 18.808) compared to interlocking nails (mean = 5.859). This makes interlocking nails superior to plates in treating tibial shaft fractures.

**Figure 3:**
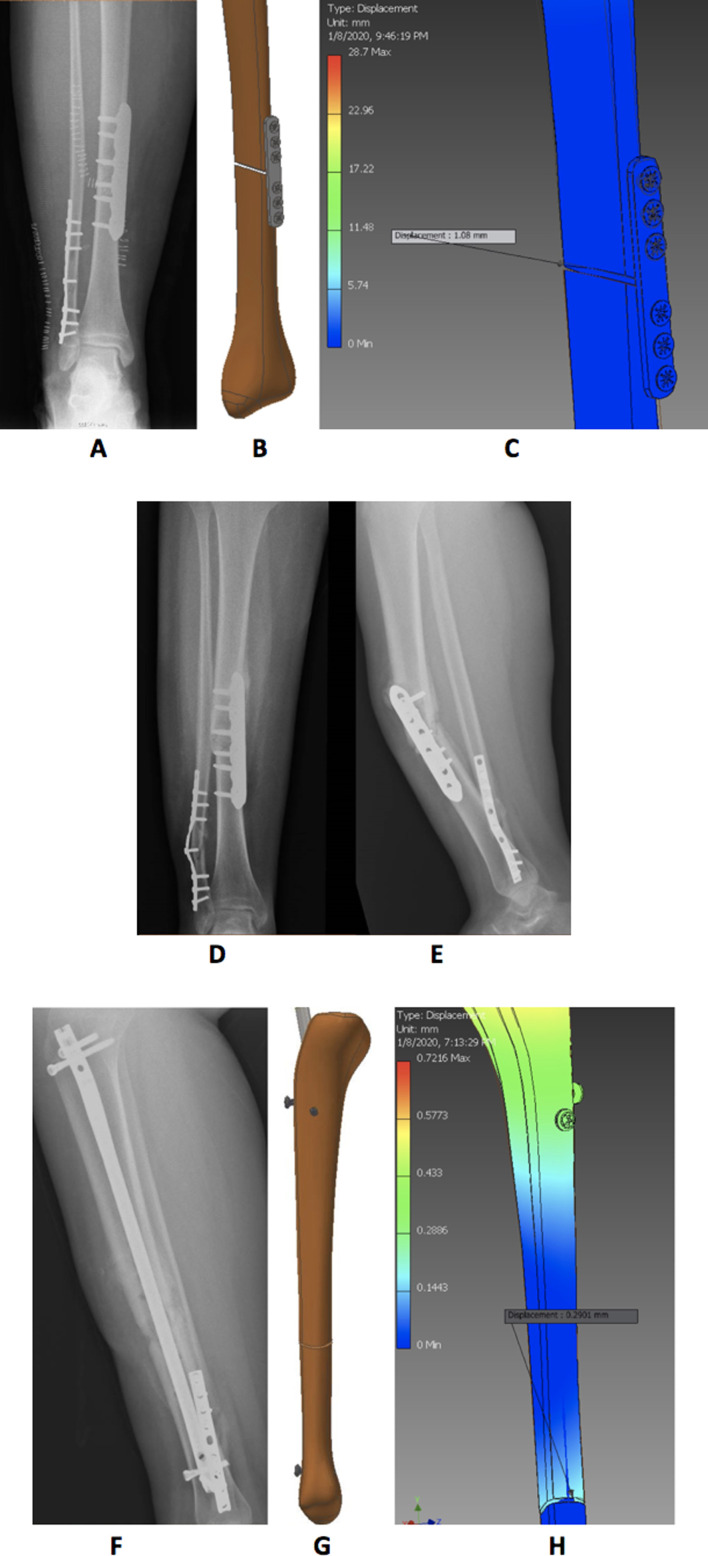
A–C: After locking plate fixation; (A) immediate postoperative X-ray, (B) 3-D model created, (C) finite element analysis of the model. D and E: X-rays after 4 weeks with failed fixation; (D) anteroposterior view, and (E) lateral view. F–H: After revision of fixation with IMN; (F) postoperative X-ray, (G) 3-D model created, (H) finite element analysis of the model.

A 25-year-old male patient in our study with a weight of 90 kg sustained a closed comminuted fracture in the tibial shaft. Open anatomical reduction and fixation were made using a locking plate. FEA revealed that the current minimum fracture gap was 3 mm. With the maximum load applied (1530 N (bodyweight + 0.7 of body weight) = 90 + 0.7 × 90 = 153 kg), the upper fragment of the fractured bone settled down according to bending in the stainless-steel plate by 1.08 mm. The strain percent in the gap after applying the load was 36%, exceeding the allowed micromotion range (2–10%). The patient started early weight-bearing against medical advice and fell down the stairs 4 weeks after surgery, which failed fixation. Revision surgery was performed using IMN, and FEA of the new X-rays showed that with the same load application, the resultant bending was 0.29 mm, and strain percent changed to 9.6% (Figure 3).

Patients with fractures of the tibial shaft that extend proximally tend to develop valgus or procurvatum malalignment if IMN is used for fixation. The use of blocking screws within the proximal fragment is one option to maintain the nail within the central axis of this fragment and avoid deformity [15].

Another 27-year-old female patient (60 kg) with a closed comminuted fracture tibia that extended to the proximal third underwent internal fixation primarily using a titanium IMN. In the postoperative radiographic assessment valgus malalignment was noticed, and FEA revealed that the current minimum fracture gap was 2.5 mm. With maximum load applied (1020 N (body weight + 0.7 of body weight) = 60 + 0.7 × 60 = 102 kg), the upper part of the fractured bone moved down according to bending in the titanium rod by 0.5 mm. The strain percent in the gap after applying the load was 20% exceeding the allowed micromotion range (2-10%). Revision surgery was done by exchanging the nail and applying blocking screws, and the FEA of the new X-rays showed that with the same load application, the resultant bending was 0.1387 mm and the strain percent changed to 5.548%.

This study included a small number of patients, managed using different types of implants with different configurations of the plates and nails. It represents the surgical technique and accuracy of a single surgical center, which may have influenced the outcome. Another limitation is that orthopedic surgeons may lack the necessary experience to deal with the FEA code. Accurate results in these studies may be more dependent on the technicians’ familiarity with the system and their ability to comprehend the design and purpose of the medical study. As a result, this could be considered a pilot study to investigate the validity of FEA in different fixation methods in tibial fractures and predict failure. This may encourage the conduct of larger-scale studies in the future.

FEA is used for the analysis of bone stress; it is the key to assessing the fracture risk, understanding bone remodeling, and designing the fracture fixation device [9]. FEA helped in determining strain percent for each patient after the surgery. Hence, patients requiring revision could be identified when the strain percent did not match the preset micromotion range.

Previous trials to use FEA in evaluating different fixation methods in tibial fractures were limited to studies performed on cadavers or tibia composites (sawbones). Hu and Hu conducted a study on 60 tibias from cadavers with created fracture models fixed with limited contact-dynamic compression plate (LC-DCP). The aim was to test different plate lengths, screw numbers, and screw positions biomechanically when compression, torsion, and bending stresses were applied. They concluded that the use of six screws through a 14-hole plate had the greatest stability, especially when 2 screws were fixed close to the fracture site and the other screws fixed at the ends of the plate [16].

Blažević et al. compared external locking plates and conventional external fixators in extraarticular proximal tibial fractures on models of the composite tibia (sawbones). These models were constructed using computer assisted design (CAD) and analyzed using FEA. They found higher stiffness in models with conventional external fixators [17].

## Conclusion

Interlocking nails showed better results for treating tibial shaft fractures, with less bending and strain percent after applying the load. Plates are more prone to bending and result in higher strain percent, leading to implant failure. The disadvantage of interlocking nailing is the possible deformity with proximally extending fractures that can be solved by blocking screws.

## Clinical trial registeration: (retrospectively registered)

Registration number: NCT04388462

Registration date: May 14, 2020
